# Biochemical and Mechanical Analysis of Occlusal and Proximal Carious Lesions

**DOI:** 10.3390/diagnostics12122944

**Published:** 2022-11-25

**Authors:** Sahar Al-Shareefi, Ali Addie, Lamis Al-Taee

**Affiliations:** 1 Department of Conservative and Esthetic Dentistry, Baghdad College of Dentistry, University of Baghdad, Baghdad P.O. Box 1417, Iraq; 2 Center of Advanced Materials, Ministry of Science and Technology, Baghdad P.O. Box 0765, Iraq

**Keywords:** caries-infected dentin, caries-affected dentin, collagen integrity, Raman microscopy, Vickers microhardness

## Abstract

A precise evaluation of caries excavation endpoint is essential in clinical and laboratory investigations. Caries invasion differentiates dentin into structurally altered layers. This study assessed these changes using Raman spectroscopy and Vickers microhardness. Ten permanent molars with occlusal and proximal carious lesions were assessed and compared at 130 points utilizing four Raman spectroscopic peaks: phosphate v1 at 960 cm^−1^, amide I (1650 cm^−1^), amide III (1235 cm^−1^) and the C-H bond of the pyrrolidine ring (1450 cm^−1^). The phosphate-to-amide I peak ratio and collagen integrity peak ratio (amide III: C-H bond) of carious zones were calculated and compared in both lesions. The former ratio was correlated to 130 Vickers microhardness indentations through lesions. The caries-infected dentin (CID) exhibited low phosphate peak, but higher amide I, III and C-H bond peaks than other zones in both lesions. The peaks in amide regions (I and III) varied in occlusal versus proximal lesions. A high correlation was found between mineral: matrix peak ratio and equivalent microhardness number within carious lesions, while the collagen integrity peak ratio was applied in proximal lesions only. Raman spectroscopy detected changes in the mineral and matrix contents within different carious zones and regions.

## 1. Introduction

Dental caries is the most common multifaceted disease with a significant societal impact that spreads worldwide [1]. The occlusal and proximal tooth surfaces are the most susceptible sites for demineralization from the bacterial acidic by-products [2]. Although the predominance of occlusal caries was higher [3], in which the bacterial accumulations receive the best protection in the deepest part of the groove-fossa system, carious progression in smooth surfaces is also popular due to the difficulty of early detection using the standard visual-tactile evaluation [4]. However, the radiographs and fiber-optic transillumination can be beneficial, but they cannot always lead to a definitive diagnosis [5]. Carious dentin is classified clinically and histologically into two main zones, superficial infected (CID) and deeper affected layer (CAD), in which the delineation between them is critical in clinical and laboratory investigations. The use of minimally invasive caries management reduces unnecessary tissue removal and the risk of pulp exposure, whilst maximizing the reparative potential of the dentin-pulp complex [6]. This includes complete excavation of the superficial, bacterially contaminated and denatured dentin while preserving the remaining harder affected dentin that can be sealed with therapeutic restorations [7]. Clinically, the discrimination between the carious zones based on tissue hardness, moisture, color, fluorescence properties and dye stainability. Additionally, carious tissue removal methods can be used to determine the endpoint, such as self-limiting burs and chemomechanical removal agents. These methods are implemented in in vitro investigations but lack sufficient clinical validation [8]. The laboratory studies assessed the mechanical properties of the carious dentin through relative changes in tissue hardness via Knoop or Vickers hardness test as a gold standard to delineate the excavation margins. However, this test is considered as an invasive, low-resolution test that damages tissues irreversibly preventing further tissue analysis [9]. Furthermore, hardness clarifies the mechanical integrity without any correlation to the biochemistry of the carious lesion. In contrast, the use non-invasive analytical technology for in vitro studies such as Raman spectroscopy will help in assessing the biochemical tissue changes during the caries process. It can be sufficiently sensitive to the differences in the mineral and organic compositions of healthy and carious dentin tissues through their specific molecular vibrational energy signatures [10]. Previous studies utilized FTIR and Raman spectroscopy to identify the spectral characteristics of mineral-to-matrix peak ratio, which represents the phosphate and amide I distributions through the carious dentin lesion [11,12,13,14]. The phosphate peak intensity is related to the mineral content, while the amide I peak intensity represents the organic component in dentin [15,16,17,18]. Thus, this correlation is regarded as a biochemical measure for dentin demineralization, in which the corresponding hardness values will help to identify the lesion characteristics in each zone. The evaluation of the collagen in carious dentin has a significant importance, since it affects the bond stability when bonded to adhesive resin restorations. The integrity of the collagen triple helix in type I collagen membrane obtained from bovine Achilles deep tendon was previously measured using attenuated total reflection Fourier transform infrared (ATR-FTIR) [19]. In this method, a peak ratio of amide III: C-H bond of the pyrrolidine ring (1235 cm^−1^:1450 cm^−1^) was used as an indicator for the collagen integrity if it is close to 1 [20]. However, this ratio was not tested on a natural dentin carious lesion using Raman microscopy, to validate using this ratio as an indicator for the collagen integrity in vitro investigations. The biochemical characterization of proximal carious lesions is scarce in the literature. The susceptibility of different tooth surfaces for caries development and progression is markedly differed in occlusal versus smooth surfaces [21], added to differences in the inflammatory pulp response in proximal lesions as compared to occlusal lesions [22]. This might necessitate an assessment of the structural changes in proximal carious lesions in comparison to occlusal in the same tooth model. Accordingly, this study evaluated and compared the changes in the mineral content and collagen matrix in different carious zones of occlusal vs. proximal carious lesions using Raman microscopy. The null hypotheses: (i) there were statistically no significant changes in the mineral and matrix contents of demineralized carious lesions (CID and CAD) from sound dentin in each lesion and between lesions, (ii) the collagen integrity ratio (Amide III: CH- bond of the pyrrolidine ring) cannot be applied for a natural carious dentin lesion, and (iii) there was no statistical correlation between Raman peak ratio of phosphate ν1:amide I ratio and the equivalent VHN in occlusal and proximal carious dentin lesions.

## 2. Materials and Methods

### 2.1. Sample Preparation for Raman and Hardness Measurements

**Figure 1:**
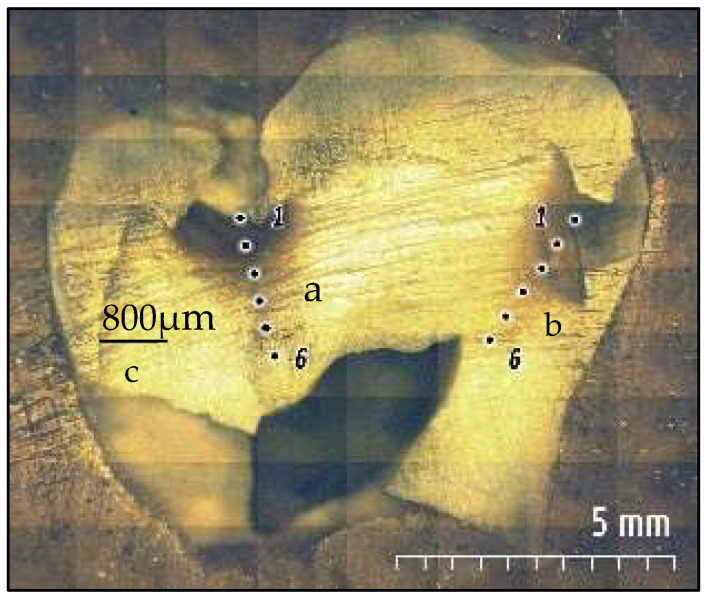
The prepared lesion hemi-section was firstly analyzed using confocal Raman microscopy followed by Vickers microhardness indenter across the lesion. The assessment started from the enamel–dentin junction through the lesions towards the pulp in a straight path occlusally (a) and an oblique path proximally (b). A control measurement was taken in an area of clinically sound dentin (c) in the same sample. Six measurement areas were taken in each lesion with a 500 μm distance between the examined points.

Ten permanent, freshly extracted human 1st molars were collected using an ethics protocol approved by the health research committee (Ref No. 285521, 31 March 2021). The teeth showed two active carious dentin lesions; occlusal and proximal at the contact area and above the cementoenamel junction. The lesions showed score 4 following the international caries detection and assessment system (ICDAS) [23], in which the lesion extended halfway through the dentin without pulp exposure, then were stored in distilled water in a cold cabinet (+4 °C). Samples were hemi-sectioned longitudinally (Isomet 1000, Buehler, Lake Bluff, IL, USA) using a water-cooled diamond blade (330-CA/RS-70300, Struers, Detroit Rd. Westlake, LLC, Cleveland, OH, USA), and then embedded in epoxy resin molds. The surfaces were polished under running water using a polishing machine (Laryee Technology, China), and silicon waterproof papers in a sequential pattern (P1200 for 10 s, P2500 for 10 s, and P4000 for 4 min) [13] to gain flat and smooth surfaces for accurate measurements. A small steel round rotary bur was used to create a reference dot at the enamel–dentin junction occlusally and proximally. These dots are visible under Raman spectroscopy and Vickers microhardness with a 500 μm distance between the examined points, as shown in Figure 1.

### 2.2. Raman Spectroscopy

A high-resolution confocal Raman microscope (Senterra, Bruker Optics, Ettlingen, Germany) operating in line scan mode was used to scan the carious lesions and sound dentin. A total of 130-point scans were made over twenty carious lesions (*n* = 13 per tooth). The distance between the scanned points was 500 μm, and it was controlled using a programmable sample stage with a 1μm resolution. Spectra acquisition was performed using a 785 nm near-infrared diode laser and a 400 line/mm diffraction grating. An Olympus 20X/0.40 NA objective lens was used to focus the laser on the sample surface with a spot size of about 5 μm. The sectioned tooth was mounted on the sample stage using plastic molding putty, and the Raman spectra for carious lesions and sound dentin were measured over the range of 200–3600 cm^−1^ with 100 mW of laser power on each point. The integration time for each spectrum was typically 30 s, with three accumulations. Spectrum acquisition was conducted using the image stitching technique to collect a large area of the mounted tooth (Figure 1). Baseline correction was performed by Raman processing software (OPUS, Bruker Optics, Germany). After acquisition and spectra processing, four Raman spectroscopic peaks were identified. The phosphate peak intensity v1 vibration at 960 cm^−1^, amide I peak intensity at 1650 cm^−1^, amide III at 1235 cm^−1^ and C-H bond of the pyrrolidine ring at 1450 cm^−1^. Peak height intensities were calculated and averaged in both lesions (occlusal and proximal) and the control point (sound dentin). The inorganic to organic ratios of dentin components were assessed from the band intensities of phosphate V1 at (960 cm^−1^) to amide I (1650 cm^−1^). While the collagen integrity at each zone was assessed by calculating the absorbance ratio of 1235 cm^−1^:1450 cm^−1^ (*n* = 2 per zone, then averaged).

### 2.3. Vickers Microhardness

The microhardness of demineralized dentin zones (caries-infected, caries-affected) and sound dentin was measured using the Vickers microhardness tester (TH714, Obsnap Instruments Sdn Bhd, Selangor, Malaysia). A square-based pyramid diamond-shaped indenter was used, with a load of 300 gf for 15 s [24]. A total of 130 indentations (*n* = 13 per lesion) were made in the same straight path line that previously assessed by Raman microscopy. These indentations started from the enamel–dentin junction occlusally and proximally toward the pulp, with 500 μm interval between the examined points (Figure 1). Then, the Vickers hardness number was recorded automatically using the manufacturer’s software.

### 2.4. Statistical Analysis and Spectral Correlation Processing with Hardness Measurements

The statistical analysis was performed using SPSS software version 25 (IBM, Chicago, IL, USA). Shapiro–Wilk test was used to evaluate the normality of data distribution. The data were statistically analyzed using one-way ANOVA followed by Tukey post hoc multiple comparisons (*p* > 0.05) regarding the intensities of four Raman peaks (A.U.) and Vickers microhardness number (VHN) in each zone per lesion. The independent t-test (Minitab 14, Minitab LLC, Chicago, IL, USA) was performed for pairwise comparisons between groups (α = 0.05) to assess the differences in these data between the occlusal and proximal lesions at each zone. Additionally, Pearson’s correlation coefficient test was used to explore if there is a correlation between Raman mineral: matrix peak ratio and their equivalent microhardness values (VHN) at each point per lesion. The peak ratios were determined after baseline correction in single spectra by dividing the intensity of phosphate to the amide I (960 cm^−1^/1650 cm^−1^).

## 3. Results

### 3.1. Biochemical Analysis of Sound and Demineralized Zones in Occlusal and Proximal Lesions

The relative Raman band intensities (Mean ± SD) with statistical correlations in caries-infected, caries-affected and sound dentin zones of the occlusal and proximal carious lesions are shown in Table 1. All dentin layers (sound and demineralized) showed the evidence of the four characteristic peaks; the phosphate peak intensity (symmetric P-O stretching mode, v1-PO) at 960 cm^−1^ which represents the inorganic part of dentin, the amide I peak intensity at 1650 cm^−1^, amide III at 1235 cm^−1^ and the C-H bond of the pyrrolidine ring at 1450 cm^−1^ which refer to the organic part of sound and demineralized dentin. One-way ANOVA showed a statistically significant difference between sound and demineralized (lesion) dentin regarding all Raman peaks in both lesions (*p* = 0.000). Fur-ther analysis using Tukey post hoc multiple comparisons revealed that the phosphate band at 960 cm^−1^ in both lesions was statistically the lowest in caries-infected dentin zone (CID, *p* = 0.000), but it was increased with increasing the mineral content in the caries-affected (CAD), and then sound dentin with non-significant difference between occlusal and proximal lesions in each zone (independent *t*-test, *p* > 0.05), Table 1.

**Figure 2:**
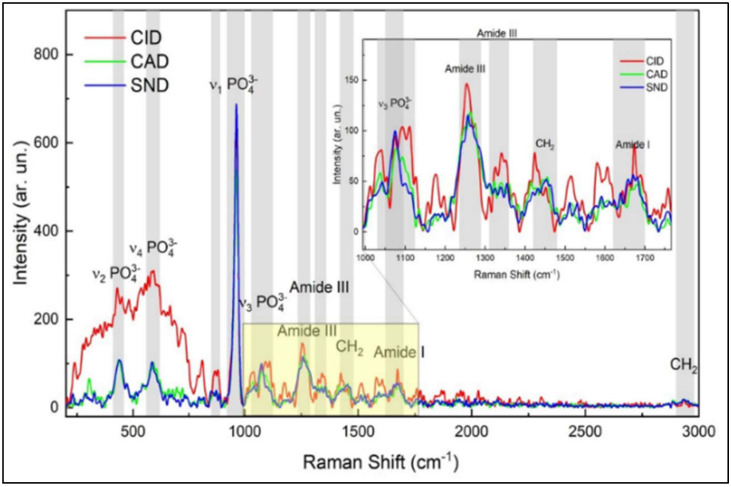
Representative Raman spectra of the caries-infected (CID), caries-affected (CAD) and sound dentin in the occlusal carious lesion. The spectra were normalized based on the peak intensities of v1-PO at 960 cm^−1^, which is the strongest signal among all Raman spectra, it is the highest in sound dentin. Amide I at 1650 cm^−1^, Amide III at 1235 cm^−1^ and C-H bond of the pyrrolidine ring at 1450 cm^−1^ are the highest in CID. The inserted figure shows the magnified, same spectra in the range from 1000 to 1700 cm^−1^.

**Figure 3:**
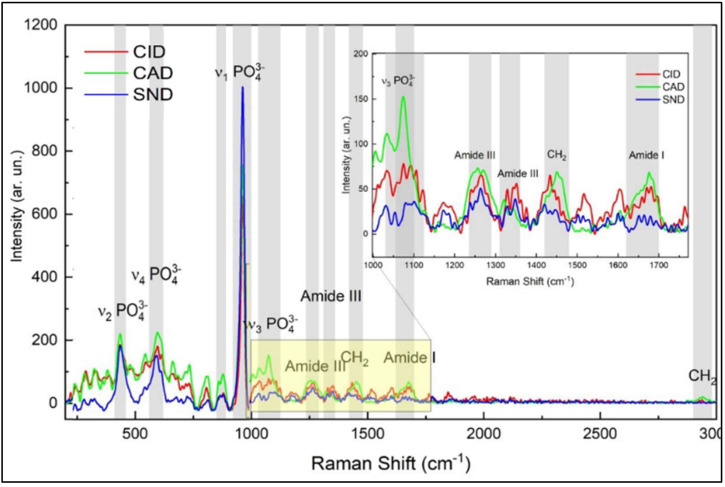
Representative Raman spectra of the caries-infected (CID), caries-affected (CAD) and sound dentin in the proximal carious lesion. The spectra were normalized based on the peak intensities of v1-PO at 960 cm^−1^, which is the strongest signal among all Raman spectra, it is the highest in sound dentin. Amide I at 1650 cm^−1^, Amide III at 1235 cm^−1^ and C-H bond of the pyrrolidine ring at 1450 cm^−1^ are higher in CID and CAD than sound dentin. The inserted figure shows the magnified, same spectra in the range from 1000 to 1700 cm^−1^.

In both carious lesions, the intensities of amide I, III and C-H bond of pyrrolidine ring peaks (1650, 1235 and 1450 cm^−1^, respectively) were statistically higher in CID, with no significant difference between CAD and sound dentin (*p* > 0.05), except the C-H bond of pyrrolidine ring peak in the proximal carious lesions which was higher in CAD than in sound dentin (*p* = 0.023), Table 1, Figure 2 and Figure 3.

By comparing the peak intensity between the occlusal and proximal carious lesions, it was found that there were statistically no significant differences in the intensity of the phosphate peak (960 cm^−1^) between both lesions (*p* > 0.05) at each zone. For the Amide I (1650 cm^−1^), the peak intensity in the caries-infected zone was higher in the occlusal lesion as compared to the proximal (*p* = 0.014), while there was statistically no significant difference in CAD and sound dentin zones between lesions (*p* > 0.05). The intensity of amide III peak (1235 cm^−1^) was significantly higher in the occlusal carious lesion in all zones (CID, CAD and sound dentin) in comparison to proximal carious lesion (*p* < 0.05). However, the intensity of C-H bond of the pyrrolidine ring peak at 1450 cm^−1^ was comparable between the occlusal and proximal lesions at each zone (*p* > 0.05).

To analyze the integrity of the collagen triple helix, a mean absorbance ratio of amide III bands at 1235 cm^−1^ and the pyrrolidine ring at 1450 cm^−1^ was calculated. In the proximal carious lesion, the mean ratio in the caries-infected dentin was 0.7 ± 0.2, while the mean ratio in CAD was 0.9 ± 0.2 that was close to sound dentin (1.0 ± 0.3). This demonstrates a lack of collagen denaturation in the CAD zone. However, this ratio was not applied in the occlusal carious lesion that showed a very high absorbance band of the amide III bands at 1235 cm^−1^ (amide III) than that of the pyrrolidine ring at 1450 cm^−1^, Figure 2.

### 3.2. Vickers Microhardness

**Figure 4:**
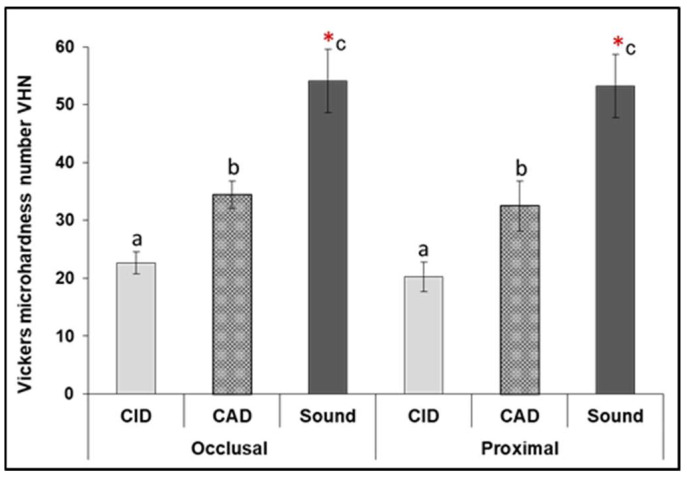
Means of Vickers microhardness number (VHN) of the sound and demineralized dentin (caries-infected and caries-affected) in occlusal and proximal lesions. (*) Statistically significant difference of demineralized dentin from sound (*p* < 0.001). Similar litters mean statistically non-significant differences (*p* > 0.05) between different dentin zones in occlusal vs. proximal lesions.

The change in the mineral content of the carious lesions was also measured via a mi-crohardness test. One-way ANOVA revealed a statistically significant difference among different dentin layers (sound vs. demineralized, *p* = 0.000) with no significant difference found between occlusal and proximal lesions at each zone (independent *t*-test, *p* > 0.05). Further analysis by Tukey post hoc multiple comparisons test showed that there was a marked reduction in VHN in CID at occlusal and proximal lesions (20.2, 22.6, respectively), which was significantly higher in CAD (34.4, 32.5, respectively, *p* = 0.000), followed by sound dentin (54.1, 53.2, respectively, *p* = 0.000), as shown in Figure 4.

### 3.3. Raman Spectral Correlation with Vickers Microhardness (VHN)

**Figure 5:**
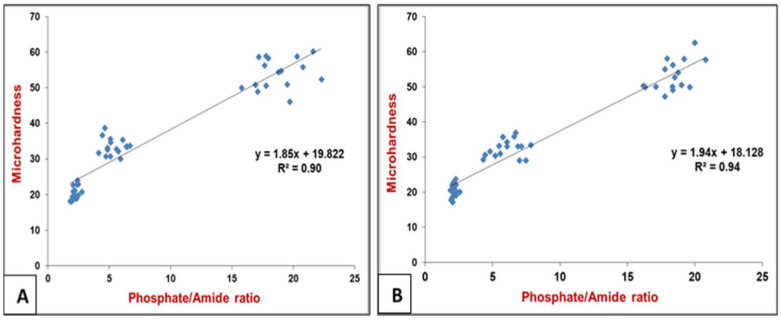
A scatter plot and a regression line (R) demonstrating the presence of a logarithmic regression relationship (*p* < 0.000) between the Raman peak ratios (phosphate v1: amide I) and Vickers microhardness (VHN) in the sound and demineralized dentin (CID and CAD) in the occlusal lesion (**A**) and proximal lesion (**B**). The peak ratio is lower in CID with reduced mineral contents which gradually increased towards sound dentin (more phosphate v1).

The peak ratios of phosphate ν1: amide I of the selected points in each carious lesion (occlusal and proximal) were calculated and plotted against Vickers hardness numbers (VHN) as a scatter diagram, as shown in Figure 5. The coefficient of determination was calculated across the assessed points in the occlusal and proximal lesions, in which the R^2^ = 0.90 and 0.94, respectively, (*p* = 0.000). The statistically significant high correlation between peak ratio and VHN in both lesions indicates that they showed a logarithmic regression relationship which enable the calculation of the tissue hardness when the peak ratio was measured.

## 4. Discussion

The improved understanding of the caries process and biology of the dentin-pulp defense and the regenerative responses encouraged the application of minimally invasive caries removal rather than the traditional surgical excavation approach. This approach relies on accurate caries diagnosis, then identifying the excavation endpoint to exclude the irreversibly caries-infected dentin while preserving the remineralizable caries-affected dentin to enhance the long-term survival of the dentin-pulp complexes. Caries invasion leads to the differentiation of dentin into zones with altered composition, collagen integrity and mineral identity. However, the understanding of these changes from the fundamental perspective of molecular structure is limited. Accordingly, this study provided a map of the biochemical changes through the different carious dentin zones in two lesion models (occlusal and proximal) utilizing Raman spectroscopy to extract the molecular information of each zone regarding the hydroxyapatite’s structural changes and collagen denaturation as the dentin transition from the superficial caries-infected zone (CID) into sound dentin. The integrity of collagen’s triple helical structure was also evaluated based on spectra collected from demineralized dentin (carious lesions) of the selected teeth. The results support the argument that there are statistically significant changes (*p* < 0.000) in the biochemical components across the carious lesions from the superficial layers towards sound dentin.

From a biochemical perspective, the mineral content was detected via the phosphate peak (PO_4_^−3^ *ν*1) at 960 cm^−1^, which was the strongest signal among all Raman spectra. This peak is referred to the degree of demineralization in natural carious enamel and dentin [25,26]. In accordance with Almahdy et al. (2012) and El-Sharkawy (2019) [18,26], the mineral content was dropped significantly (*p* = 0.000) in CID, but higher in CAD towards sound within the same sample with no significant differences in the intensities between the occlusal and proximal carious lesions (*p* > 0.05) at each zone. The presence of amorphous Ca/P provides a local ion-rich environment, which is favorable for in situ generation of prenucleation clusters, succeeding further dentin remineralization [27]. Accordingly, the first stated hypothesis was partially rejected as the mineral content represented by the phosphate peak intensity differed among different dentin zones (sound and demineralized) but was comparable between occlusal and proximal lesions.

Amide I peak (1650 cm^−1^) is the most prominent organic component of dentin, predominantly type I collagen [28]. This band is assigned to Y8a tyrosine side chain of solution-phase collagen representing the secondary structure of proteins [29]. It was used in many studies to detect changes in the molecular structure of collagen [16,30]. The amide I content was higher in CID in both lesions, and decreased in CAD and sound dentin, as shown in Figure 2 and Figure 3. It was also higher in occlusal than proximal carious lesion. This might be attributed to the higher proteins in the infected layer than sound dentin [31]. The intensity of amide I is correlated to the non-reducible cross-links in collagen [32], which means that there is an apparent change in the molecular structure of collagen in the superficial CID layer in both lesions. This alteration in collagen is correlated to the presence of esters in the carious tissues [17] derived from the bacterial lipid components. This will promote esterification of the carboxylic side-chains of aspartate and glutamate residues catalyzed by the acidic environment (lactic acid) [33]. The closer proximity in the intensity of this band between CAD and sound dentin in both lesions might indicate the importance of preserving this layer since the organic matrix in dentin regulates the growth and maturation of apatite crystals and thus the mineralization process.

The organic component of carious dentin has a major role in the progression of carious development [16,34]. It represents the integral component of the mineralized tissue, but after demineralization, they become exposed and altered structurally [17]. The demineralization process in carious dentin can be assessed by the difference in the mineral/protein band ratio of sound and carious dentin. The band ratio of amide I at ∼1650 cm^−1^, which is the most prominent organic moiety in dentin, and the phosphate ion at ∼960 cm^−1^ was used in spectroscopic studies [13,14,17,35] to analyze the distribution of mineral content in the dental tissues. Using this band ratio, the Raman peaks graphs showed the heterogeneous nature of caries dentin in terms of mineral distribution with lower values for carious-infected dentin compared to CAD and sound dentin. This band ratio increased by increasing the mineral content in the caries-affected zone followed by sound dentin in both lesions, as shown in Figure 5.

The integrity of the collagen triple helix was evaluated by assessing the ratio of the absorbance bands of amide III at ∼1235 cm^−1^ and CH2 scissoring at ∼1450 cm^−1^ (stereochemistry of the pyrrolidine rings). The former peak is sensitive to the presence of secondary structure of collagen, while the latter is independent of the ordered structure of collagen [20]. The ratio indicates collagen with an integral triple helical structure. If the value is close to 1, but if the ratio <0.8, it means that there is a breakdown of the triple helix in this zone, such as gelatin [19,20]. The mean ratio of amide III: CH2 (1235 cm^−1^:1450 cm^−1^) was 0.7 in CID in the proximal lesion, 0.9 in CAD that was close to sound dentin (1.0) which indicates the integrity of collagen fibers that is essential for the remineralization processes. This supported that the infected layer with comprised denatured collagen, loses the potential for remineralization and must be removed. Conversely, the affected layer, that is partially demineralized and remineralizable with collagen fibrils retaining their natural structure around intact dentinal tubules, is to be preserved to maximize the reparative potential and reduce the risk of pulp exposure [36]. However, this ratio was not achieved in the occlusal carious lesion model. This is due to the higher absorbance of amide III than CH2-CH- bands in the CID zone, as shown in Figure 2, with statistically no significant difference between CAD and sound dentin (*p* > 0.05). This is referred to as the increased aliphatic side-groups of various amino acid residues in the infected tissues in these lesions which promote the demineralization process [37]. The difference between the occlusal and proximal lesions indicate that this ratio cannot be applied for all carious studies due to the variation in collagen denaturation in various tissues and regions. This is due to the variety in depth, degree of organic matrix destruction and loss of mineral content which affect tissue response to different therapeutic materials in an attempt to repair and remineralize the damaged tissues. Accordingly, the second stated hypothesis was partially accepted.

The caries-infected dentin showed higher accentuated amide I and III peaks in occlusal lesions in comparison to proximal carious lesions, which might be due to the higher aliphatic content. This is also might be attributed to the fact that the dentin subjacent to proximal enamel caries forms mineral crystals obliterating the main transport pathways (dentinal tubules), creating sclerotic dentin close to the enamel–dentin junction even before the demineralization reaches the enamel–dentin junction [38]. However, a histological study [39] reported a higher frequency of deep dentin demineralization (>50%) in proximal lesions than occlusal lesions under microradiography with contrast (MRC). This might suggest the presence of facilitated transport pathways for dentinal fluid in dentin underlying the proximal enamel lesion, since the tubules in sclerotic dentin are partially obliterated [40]. This induces a facilitated transport of dentinal fluid into the pores of carious enamel mixed with enamel fluid, and then with bacterial biofilm fluid, and thus promoting the carious progression in proximal lesions.

The microhardness values remain the gold standard for characterizing sound and demineralized dentin tissues in most in vitro studies. It is correlated to the clinical mechanical excavation procedures, and also can predict the behavior of dentin/restoration interfaces, as the regional differences in tissue hardness can alter the distribution of stresses along the interface and thus affecting the preferential location of failures. Additionally, it represents a direct measure of the hydroxyapatite (HAp) crystals present in dental hard tissues. This study showed a gradual increase in tissue hardness from the superficial carious layer towards the deeper area of the carious lesions towards sound dentin (*p* = 0.000). The caries-infected dentin showed the lowest VHN values in both lesions <25, Figure 4, which is attributed to the greater dissolution of HAp compared to the deeper CAD layer (<40) that has the potential to be repaired when sealed with self-adhesive restorations. However, the carious dentin lesion in vivo progresses in a continuous wave rather than distinct zones with gradual transitions in histology and bacteriology from enamel to pulp, it is perhaps rather simplistic to consider the lesion as three distinct zones as described above, but clinically, this analysis has merit because it allows for more reliable, efficient and reproducible operative treatment [41].

Raman can potentially evaluate the carious dentin non-destructively, as an alternative to the invasive, low-resolution hardness tests. This study found statistically significant correlations (R^2^ = 90, 94%; *p* = 0.000) between Raman peak ratio (phosphate v1: amide I) and Vickers microhardness number (VHN) in both lesions as shown in Figure 5. This Raman ratio was selected since the mineral content is considered as an indicator of the inherent physical properties of hard tissues. This correlation supports the use of this non-invasive high-resolution technique for chemical characterization in in vitro hard tissue studies rather than the physical tissue microhardness. This has important implications for the tissue pathogenesis and the minimally invasive treatment modalities. Consequently, the third stated hypothesis was rejected.

## 5. Conclusions

Based on the overall results of this study, the use of Raman spectroscopy and subsequent spectra analyses are highly useful for probing the molecular structure of carious dentin in various zones and regions. Raman detected structural changes in the inorganic and organic components of demineralized dentin in comparison to sound dentin in various regions. In which the mineral distribution was the lowest in the caries-infected dentin zone with no differences between occlusal and proximal lesions, whilst the collagen cross-link and triple helical structure were altered differently in carious zones and lesions. The presence of a statistical correlation between the Raman phosphate to amide I peak ratio with VHN supports the use of this non-invasive high-resolution technique for chemical characterization in in vitro studies. This will help further understanding of carious progression and assessing the remaining dentin tissues after different caries removal techniques following the minimally invasive approaches.

## Figures and Tables

**Table 1:** Raman band intensities A.U. (Mean ± SD) of caries-infected, caries-affected and sound dentin in occlusal and proximal carious lesions.

Raman Peaks	Carious Zones (*n* = 10 per Zone)	Mean of Peaks Intensity (Occlusal Lesion) (Mean ± SD)	Mean of Peak Intensity (Proximal Lesion) (Mean ± SD)
Phosphate peak v1-PO (960 cm^−1^)	CID	89.0 ± 11.3 ^a^	79.6 ± 7.7 ^a^
	CAD	344.4 ± 95.1 ^b^	369.6 ± 84.6 ^b^
	Sound	2105.3 ± 112.1 *^c^	2156.4 ± 122.7 *^c^
Amide I (1650 cm^−1^)	CID	84.0 ± 7.1 *	68.5 ± 7.2 *^
	CAD	62.0 ± 9.6 ^d^	60.5 ± 10.9 ^d^
	Sound	60.6 ± 9.9 ^e^	51.81 ± 10.2 ^e^
Amide III (1235 cm^−1^)	CID	112.1 ± 5.3 *	69.8 ± 3.7 *^
	CAD	80.4 ± 5.7	60.4 ± 6.0 **^**
	Sound	78.5 ± 11.5	56.7 ± 7.9 **^**
C-H bond of pyrrolidine ring (1450 cm^−1^)	CID	88.9 ± 4.9 *^f^	85.3 ± 3.9 *^f^
	CAD	74.9 ± 5.5 ^g^	80.0 ± 5.7 ^g^
	Sound	70.7 ± 8.6 ^h^	68.1 ± 9.8 ^h^

(*) significant difference between sound and demineralized dentin (caries-infected or caries-affected), one-way ANOVA test and Tukey post hoc tests (alpha level of 0.05). (**^**) significant difference between occlusal and proximal carious lesion (Independent *t*-test, *p* < 0.05). Similar letters in rows indicate no significant differences between lesions (*p* > 0.05).

## Data Availability

Not applicable.
